# Predictors of shunt insertion in patients with aneurysmal subarachnoid haemorrhage—a single-centre retrospective analysis

**DOI:** 10.1007/s00701-024-05926-1

**Published:** 2024-01-19

**Authors:** Tenna Capion, Alexander Lilja-Cyron, Markus Harboe Olsen, Kirsten Møller, Marianne Juhler, Tiit Mathiesen

**Affiliations:** 1 Department of Neurosurgery, The Neuroscience Centre, Copenhagen University Hospital Rigshospitalet, Copenhagen, Denmark; 2 Department of Neuroanaesthesiology, The Neuroscience Centre, Copenhagen University Hospital Rigshospitalet, Copenhagen, Denmark

**Keywords:** Subarachnoid haemorrhage, Aneurysm, Aneurysmal subarachnoid haemorrhage, Hydrocephalus, External ventricular drain, EVD, Weaning, Shunt, Predictive factors

## Abstract

**Background:**

No standard has been established regarding timing and choice of strategy for discontinuation of external ventricular drainage (EVD) in patients with aneurysmal subarachnoid haemorrhage (aSAH), and little is known about the importance of clinical variables. A proportion of the patients who initially pass their discontinuation attempt return with delayed hydrocephalus and the need of a permanent shunt. Early differentiation between patients who need a shunt and those who do not would facilitate care.

We conducted a retrospective analysis on patients with aSAH and an EVD to search significant differences in treatment and clinical variables between patients who received a permanent shunt during initial hospitalization or after readmission, and those who never received a shunt.

**Methods:**

We included 183 patients with aSAH who received an EVD over a 4-year period between 2015 and 2018 and divided them into three groups: those who received a shunt during primary admission, those who were readmitted for delayed hydrocephalus and received a shunt, and those who never needed a shunt. Between these groups, we compared selected clinical variables as well as outcome at discharge and after 6 months. Additionally, we assessed the ability of a shunt dependency score (SDASH) to predict the need for permanent drainage in the patients.

**Results:**

Of 183 included patients, 108 (59%) ultimately received a ventriculoperitoneal (VP) shunt. Of these, 89 (82%) failed discontinuation during the primary admission and received a permanent shunt before discharge from the neurosurgical department. The remaining 19 (18%) were discharged after successful discontinuation, but subsequently developed delayed hydrocephalus and were admitted for shunt placement a median of 39 (range: 18–235) days after ictus. Ninety-four patients were discharged after successful discontinuation of the EVD, consisting of those who never developed the need for a permanent shunt and the 19 who were readmitted with delayed hydrocephalus, corresponding to a 20% (19/94) readmittance rate. Clinical variables such as drainage volume or discontinuation strategy did not differ across the three groups of patients. The SDASH score failed to provide any clinically useful information regarding prediction of shunt placement.

**Conclusion:**

In this study, clinical variables including use of the predictive score SDASH predicted neither the overall need for nor the timing of shunt placement after aSAH. The homogeneous distribution of data between the three different groups renders strong independent clinical predictive factors unlikely. Thus, attempts to predict a permanent shunt requirement from these variables may be futile in these patients.

## Introduction

Treatment for acute hydrocephalus following aneurysmal subarachnoid haemorrhage (aSAH) with an external ventricular drain (EVD) has been standard care since the mid-1980s [6]. However, when and how to discontinue the EVD have proven challenging. This is in part because 30–40% of patients with an EVD following aSAH will remain dependent on CSF diversion after the initial phase [1, 2, 18] but are hard to identify. Thus, premature removal of the EVD may increase the risk of subsequent hydrocephalus, render EVD re-insertion followed by VP shunt placement necessary, and delay rehabilitation and recovery [23]. Conversely, prolonged discontinuation and multiple discontinuation attempts have been associated with complications; primarily ventriculitis, unfavourable functional outcome, and a higher risk for ventriculoperitoneal (VP) shunt placement [1, 2, 20]. To further complicate the matter, some patients who initially pass the discontinuation attempt return with delayed hydrocephalus and the need of a VP shunt [1, 2, 16]. These patients are suspected of having higher mortality at 6 and 12 months than patients who never needed a VP shunt after the initial EVD treatment [1].

There is little consensus regarding the timing and choice of strategy for EVD discontinuation [4]. This is primarily due to limited information from randomized clinical trials [15]. However, clinical variables such as number of discontinuation attempts, CSF protein concentration, CSF drainage production during weaning and before shunt placement may be important to identify patients at risk of failing discontinuation [2, 17–19]. Indeed, prediction scores such as the Chronic Hydrocephalus Ensuing from SAH Score (CHESS) [12] which assesses risk of shunt dependency after aSAH on a scale from 0 to 8 based on clinical and radiographic measures; the Barrow Neurological Institute (BNI) score [25] which was initially developed with the aim of predicting symptomatic vasospasm after SAH based on thickness of SAH on CT scan; and the more recent and refined Shunt Dependency in aSAH (SDASH) score [8] all attempt to predict “need for shunting”. However, these scores have not gained traction in clinical use even though the CHESS has been externally validated [27].

In an attempt to identify important clinical variables that might help predict the need for permanent CSF drainage, we retrospectively analysed data from patients with aSAH who had EVD inserted due to acute hydrocephalus from our institution. Accordingly, we compared clinical variables between three groups of patients, i.e., those who underwent shunt placement either immediately or after readmission following initially successful weaning, respectively, and those who never received a shunt. Furthermore, we assessed the predictive ability of the SDASH score. We hypothesized that CSF drainage production or weaning strategy is an indirect marker of CSF clearance and thus would predict the need for permanent drainage.

## Methods

### Study population

This was a retrospective analysis of data from patients acutely admitted to the Department of Neurosurgery, Copenhagen University Hospital, Rigshospitalet from 01.01.2015 to 31.12.2018. Inclusion criteria were (1) age ≥ 18 years of age, (2) diagnosis of aSAH, (3) treatment with an EVD during the primary admission for aSAH. Pre-defined study parameters were systematically registered from electronic patient charts. Patients were excluded from the study if they died within the first 7 days after ictus. The inclusion period was limited to ultimo 2018 since the DRAIN clinical trial started including patients in 2019. In DRAIN discontinuation of the EVD was carried out according to allocated treatment groups (ClinicalTrials.gov identifier: NCT03948256) [5].

### Management of aSAH and EVD treatment

Patients diagnosed with aSAH underwent either computed tomography angiography (CTA) or digital subtraction angiography (DSA) to confirm presence of an aneurysm as the source of the haemorrhage. Subsequently, aneurysms were secured by either endovascular treatment or surgical clipping. Placement of an EVD was decided by the physician in charge based on acute hydrocephalus verified by a CT scan, clinical deterioration (headache, nausea, and/or decline in Glasgow Coma Score (GCS)), or both. Accordingly, not all patients might have had radiologically verified acute hydrocephalus, as the EVD might have been placed on a basis of clinical deterioration or signs of increased ICP. This proportion was examined using the relative bicaudate index in which a level > 1 on the admission CT scan confirms radiological hydrocephalus.

As per department routine, EVDs were kept open at a drainage resistance of 20 cm H_2_O before securing the aneurysm, after which resistance was lowered to 10 cm H_2_O as a standard. The EVD was either gradually weaned or promptly closed at the discretion of the treating physician. In general, our patients received a shunt if repeated attempts of weaning from the EVD resulted in clinical neurological deterioration (with or without concomitant intracranial pressure increase), which was reversed by reopening or lowering the EVD. The number of discontinuation attempts was subject to individualized decisions of the clinical picture and may differ from patient to patient.

Here, “prompt closure” refers to direct closure of the EVD without a preceding weaning period, while ‘gradual weaning’ refers to stepwise increase from 10 cmH_2_O upwards of drainage height over several days. Drainage resistance was lowered or the EVD reopened in case of ICP increase to > 20 mmHg for ≥ 20 min, clinical deterioration (intolerable headache, nausea, or a decline in GCS), or hydrocephalus verified by a CT scan. Following EVD closure, whether this was done promptly or after weaning, the patient was monitored for 24 to 48 h after EVD closure (prompt or following weaning), and the EVD was then removed if the condition becomes stable.

The decision to insert a permanent VP shunt during the primary admission was based on repeatedly failed EVD discontinuation attempts and/or CT-verified hydrocephalus combined with a decline in GCS. Following discharge, shunt implantation was decided by symptoms related to delayed hydrocephalus (gait changes, lack of progress in rehabilitation, etc.). Placement of a VP shunt was decided by the treating physician as departmental guidelines were not present at the time.

SDASH score.

The SDASH score is a combined scoring tool to predict shunt-dependent hydrocephalus after aSAH [8]. It contains three clinical factors which adds up to a score between 0 and 4 points with equivalent rising risks of receiving a permanent shunt. The following clinical parameters are included in the SDASH score: presence of acute hydrocephalus = 2 points; Barrow Neurological Institute (BNI) score ≥ 3 = 1 point; and modified Fisher grade 4 = 1 point. We substituted BNI Score ≥ 3 with modified Fisher grade 4 for pragmatic purposes.

### Data management

Electronic patient charts were reviewed retrospectively, and data was entered into an online REDCap (Research Electronic Data Capture) database [10]. GCS was recorded at the time of admission and discharge from the Department of Neurosurgery [13]. Functional outcome (modified Rankin Scale, mRS) was measured at the time of discharge from the Department of Neurosurgery and 6 months after ictus [3]. Acute hydrocephalus was defined as a relative bicaudate index > 1 on the latest CT-scan before placement of the EVD as defined by Van Gijn et al. [11]. Ventriculitis was defined as the use of intrathecal antibiotics for an EVD-related infection. We recorded clinical data regarding the initial treatment phase (time of ictus, clinical status, time of EVD placement, type of aneurysm treatment, Hunt and Hess scale, and modified Fisher grade), EVD management (time of placement and removal, drainage production, strategy for discontinuation, and time of shunt placement), and follow-up (clinical status at discharge and at 6 months after ictus).

Patients were divided into the following groups:

- Patients who underwent successful discontinuation during their admission and were discharged with no subsequent need for permanent drainage—i.e., never received a shunt.
- Patients who failed discontinuation and underwent shunt placement during the admission for aSAH—i.e., received a shunt during primary admission.
- Patients who underwent discontinuation and were discharged but subsequently readmitted due to recurring (delayed) hydrocephalus and underwent shunt placement at this time—i.e., received a shunt after primary admission.

### Statistical analyses

Continuous variables are given as percentage, mean, and median and were compared between groups using Wilcoxon signed-rank test or Fishers exact test. *P* values < 0.05 were considered statistically significant. Furthermore, evaluation of the SDASH score was done by comparing shunt rates for each SDASH score, constructing a receiver operating characteristics (ROC) curve with subsequent assessment of area under the curve. Data were analysed using R v 3.2.3 (*R Core Team, Vienna, Austria*).

## Results

During the 4-year inclusion period, 204 patients with aSAH and an EVD were included. Of these, 20 (10%) died within the first 7 days after ictus, and thus a discontinuation process of the EVD was never initiated. Furthermore, one patient’s electronic medical chart could not be accessed, leaving a total of 183 patients for analysis. Patient characteristics can be seen in Table 1**.**

**Table 1:** Patient characteristics

	Shunted (*n* = 108)	Never shunted (*n* = 75)	*P*
Female (% (*n*))	71.3 (77)	54 (72)	1
Age (mean (range))	63.5 (34–88)	57.6 (30–89)	0.001
GCS on admission (median (range))	6 (3–14)	8 (3–13)	< 0.001
Intubated at admission (% (*n*))	51.9 (56)	29.3 (22)	0.004
Hypertension (% (*n*))	39.8 (43)	33.3 (25)	0.437
Treatment of aneurysm (% (*n*))			0.031
Surgical	42.6 (46)	30.7 (23)	
Endovascular	57.4 (62)	65.3 (49)	
N/A	0	4 (3)	
Aneurysm location (% (*n*))			0.081
ICA	17.6 (19)	14.7 (11)	
ACA/ACOM	45.4 (49)	38.7 (29)	
MCA	14.8 (16)	10.7 (8)	
Basilar	19.4 (21)	20 (15)	
Vertebral/PICA	1.9 (2)	10.7 (8)	
Pericallosa	0.9 (1)	4 (3)	
N/A	0	1.3 (1)	
Hunt Hess (% (*n*))			0.001
1–3	53.7 (58)	74.7 (56)	
4–5	44.4 (48)	22.7 (17)	
N/A	1.9 (2)	2.7 (2)	
Modified Fisher Grade (% (*n*))			0.009
0–3	36.1 (39)	53.3 (40)	
4	63.9 (69)	45.3 (34)	
N/A	0	1.3 (1)	

### Shunt insertion

One hundred and eight patients (59%) ultimately received a VP shunt. Of these, 89 (82%) failed the discontinuation during the primary admission and received a shunt before discharge from the neurosurgical department. The remaining 19 (18%) were discharged after successful discontinuation but subsequently developed delayed hydrocephalus and were admitted for shunt placement at a median of 39 (range: 18–235) days after ictus. Seventy-five patients never underwent shunt placement (75/183, 41%) **(**Table 2**).**

**Table 2:** Clinical variables related to EVD discontinuation

	Shunted (*n* = 108)	Never shunted (*n* = 75)	*P*
Time from EVD placement to first discontinuation attempt ((mean days (range))	9.6 (1–24)	8.7 (1–17)	0.29
Output/ml (mean (range))	230 (70–423)	214 (50–410)	0.099
Resistance of 10 cmH2O ((% (*n*))	58.3 (63)	58.7 (44)	1
Time from EVD placement to last discontinuation attempt (mean days (range))	15.0 (2–32)	13.1 (5–28)	0.013
Output/ml (mean (range))	176.8 (20–475)	166 (40–400)	0.677
Resistance of 10 cmH_2_O (% (*n*))	39.8 (43)	46.7 (35)	0.367
Gradual weaning (% (*n*))	76.9 (83)	74.7 (56)	0.658
Hydrocephalus (RBCI > 1) (% (*n*))	66.7 (72)	70.7 (53)	0.645
Ventriculitis (% (*n*))	35.2 (38)	36 (27)	0.875

### Functional outcome

Favourable outcome (mRS 0–2) at time of discharge was observed in 22 (31%) and 6 (35%) among the groups shunted during and after primary admission, respectively. Six months after ictus, the share of patients with mRS 0–2 had increased to 40 (57%) and 13 (77%), respectively. All-cause mortality at 6 months was 12/89 (14%) and 0/19, respectively **(**Table 3**)**.

**Table 3:** Data on shunt insertion and outcome

	Shunted during primary admission (*n* = 89)	Shunted after primary admission (*n* = 19)	*P*
Time to shunt (median (range))	17 (8–41)	39 (18–235)	0.001
Mortality within 6 months (% (*n*))	13.5 (12)	0	0.171
GCS at discharge (median (range))	14 (6–15)	14 (9–15)	0.133
mRS 0–2 at discharge (% (*n*))	31.4 (22)	35.3 (6)	0.569
mRS 0–2 at 6 months (% (*n*))	57.1 (40)	76.5 (13)	0.212

### Clinical variables related to EVD discontinuation

Neither timing of discontinuation nor CSF drainage production differed between patients who received a shunt and those who did not **(**Table 2**)**. Timing of the discontinuation shows the number of days between EVD placement and the first and the last discontinuation attempt. Gradual weaning was the primary discontinuation strategy in 77% and 75% of patients in the shunted and non-shunted group, respectively. The prevalence of hydrocephalus on the initial CT scan, time from EVD placement to shunt placement, the level of drainage resistance (cm H_2_O) of the EVD prior to each discontinuation attempt, and share of ventriculitis also did not differ between groups (Table 2).

### SDASH prediction score

We applied the SDASH prediction score on our data to assess which proportion of patients with each score received a shunt. Table 4 shows that the risk of receiving a shunt was comparable across all SDASH scores as 27%, 25%, and 21% received a shunt despite the SDASH score increasing from 2 to 3 and 4 points, respectively. The resulting area under the ROC curve was 0.58 (95% confidence interval: 0.50–0.66) (Table 5).

**Table 4:** Application of SDASH score

SDASH score	Prediction from SDASH score	Actual number of shunted patients with this score (*n*, %)
0	2.9%	5/108, 4.6%
1	18.6%	14/108, 13%
2	40.6%	29/108, 27%
3	50%	27/108, 25%
4	76.2%	23/108, 21%
N/A	-	10/108, 9.3%

*N* = *Number of shunted patients in our cohort with each respective SDASH score*

*Prediction from SDASH score* = *the predicted share of patients with Shunt-dependent hydrocephalus, as calculated in the SDASH score*[8]

**Table 5:** The specifics for each calculated SDASH variable

	Never shunted (*n* = 75)	Shunted (*n* = 108)	
		Shunted during primary admission (*n* = 89)	Shunted after primary admission (*n* = 19)
SDASH (*n* (%))			
0	14 (18.7)	5 (5.6)	0
1	5 (6.7)	14 (15.7)	0
2	22 (29.3)	23 (25.8)	6 (31.6)
3	22 (29.3)	18 (20.2)	9 (47.4)
4	9 (12)	20 (22.5)	3 (15.8)
Missing	3 (4)	9 (10.1)	1 (5.3)
Modified Fisher Scale			
0	0	1 (1.1)	0
1	3 (4)	1 (1.1)	1 (5.3)
2	21 (28)	15 (16.9)	0
3	16 (21.3)	16 (18)	5 (26.3)
4	34 (45.3)	56 (62.9)	13 (68.4)
Missing	1 (1.3)	0	0
Hunt Hess			
1	2 (2.7)	1 (1.1)	0
2	29 (38.7)	12 (13.5)	9 (47.4)
3	25 (33.3)	32 (36)	4 (21.1)
4	5 (6.7)	16 (18)	1 (5.3)
5	12 (16)	27 (30.3)	4 (21.1)
Missing	2 (2.7)	1 (1.1)	1 (5.3)
Acute hydrocephalus			
N	18 (24)	25 (28.1)	1 (5.3)
Y	53 (70.7)	55 (61.8)	17 (89.5)
Missing	4 (5.3)	9 (10.1)	1 (5.3)

## Discussion

In this study we investigated if clinical variables such as drainage volume, discontinuation strategy, or number of weaning attempts could be potential predictors for shunt insertion, and subsequently validated a recently published prediction score for “shunt dependency” [8]. We analysed these clinical parameters against shunt insertion (1) during the primary admission, (2) during the rehabilitation phase, and (3) not at any time. We observed extensive overlap between the three groups in terms of CSF production, discontinuation strategy, number of discontinuation attempts as well as time to discontinuation. Thus, we could not use the drainage production or strategy for discontinuation of the EVD to distinguish between the two groups of patients who received shunts during or after primary admission, and those who never received a shunt. We also added Fisher grade, aneurysm location, age, and GCS on admission, which have previously been suggested as predictors. However, even though several variables were slightly unequally distributed between the three groups, none of these reached statistically significant differences nor sufficient discrimination to support clinical decisions. The results indicate that the decision to insert of a shunt reflects qualitative clinical deliberation. Quantitative predictors should thus be looked for amongst other parameters, e.g., CSF composition [22].

### Clinical application of predictive scoring

The high rate of post-haemorrhagic hydrocephalus after aSAH is well-documented [1, 7, 15, 21]. However, rather than the decision to place an EVD, the clinical challenges of CSF diversion after aSAH are related to the subsequent discontinuation. It would be desirable to minimize the duration of external drainage and place a permanent shunt in all patients who need one as early as possible. To clarify this question, several prediction scores with the purpose of identifying exactly which patients eventually received a shunt have been published [8, 9, 12, 14, 24]. The prediction scores are typically based on clinical parameters such as Hunt Hess score, modified Fisher grade, and presence of acute hydrocephalus or an EVD [8, 12]. A high discriminatory performance has been reported for prediction scores which assign a high weight to acute hydrocephalus or “need for an EVD”, and which are applied to all patients with SAH (24). External validation of these prediction scores has been limited (24,25) and confined to consecutive cohorts of patients of all Hunt Hess grades and those with and without an EVD. This also applies to the most recently introduced score, the composite SDASH score (14). Although developed specifically for predicting shunt dependency following SAH, in the present study the area under the receiver operating curve was 0.58, indicating slightly better prediction than chance. Our findings suggest that the discriminatory performance of these scores is limited when it comes to predicting shunt insertion in patients with SAH, once an EVD has been placed.

Most studies and prediction scores claim to predict “shunt dependency”, while in fact “shunt insertion” is the traceable outcome. As shunt dependency is de facto impossible to assess “shunt insertion” is then used as a proxy for dependency without direct assertion of how dependency should be defined. The precise assessment of shunt dependency would require removal of the shunt at a designated time point to assess whether the patient clinically was in fact shunt dependent. It is believed that the resolution of blood from the subarachnoid space may diminish the need for a shunt on a longer-term scale. However, the duration of temporary shunt dependency may differ from patient to patient. Shunt insertion as an outcome is independent of functional outcome or quality of life for patients treated with a VP shunt [26]. Shunt placement is a traceable outcome marker; yet, shunt placement as well as EVD placement ultimately reflects human decision making rather than natural law. The actual decision to place the shunt is complex, and it is possible that decision making differs between surgeons and centres, with subsequent heterogeneity detectable when validation is undertaken in independent datasets.

### Shunt insertion and associations with CSF drainage, predictive scoring, and weaning data

We found a cumulative VP shunt rate of 59% in our cohort of patients with aSAH and an EVD. Placement of an EVD for acute hydrocephalus is a dominating independent risk factor for permanent shunt placement [1, 7, 20] which we replicated, but with an even higher VP shunt rate than in previous literature. The association between hydrocephalus and shunt placement is frequently reported as a primary finding, and hydrocephalus forms an important component of prediction scores such as SDASH [8]. However, identification of hydrocephalus as a risk factor cannot change practice as hydrocephalus already is the main indication for shunting. More importantly, other potential predictors have been examined. The literature describes significant associations between shunting and CSF drainage volume, size of third ventricle, and CSF protein concentration (8, 20). Here, clinical translation is dependent on effect size to allow discrimination of subpopulations, and clinical applicability has not been addressed in the original or subsequent studies (8, 20). Moreover, predictive factors have been aggregated in the form of predictive scores [8, 12, 14] such as most recently the SDASH score [8]. In our cohort, a predictive score such as SDASH did not provide clinically useful information. Each different score between 1 and 4 was associated with a shunt rate between 5 and 27%; this association could not have been used to inform clinical management.

### Readmittance for shunt placement

Shunt insertion after failure to discontinue drainage is clinically straightforward. In the acute clinical scenario where an EVD is either promptly closed or gradually weaned as a test for “shunt dependency”, failure to discontinue drainage will directly lead to insertion of a shunt. It is more relevant but probably more difficult to identify patients who would develop delayed hydrocephalus and be readmitted for shunt insertion after successful discontinuation and discharge.

Akinduro et al. and Chung et al. described readmission for shunting of chronic hydrocephalus in 15% and 11%, respectively [1, 7]. Of the total group of shunted patients, 18% (19/108) received the shunt for delayed hydrocephalus, meaning that they succeeded EVD discontinuation during primary admission and were initially discharged. Interestingly, almost all these patients had acute hydrocephalus on their admission CT scan with a relative bicaudate index (RBCI) above 1, while only one of 19 patients who did not have acute hydrocephalus was readmitted for a shunt. Clinically, a nearly 20% (19/94) risk of readmittance for shunt placement shows a need for follow-up with scanning to identify progressive hydrocephalus and allow shunt surgery before clinical deterioration.

In connection hereto, absence of acute hydrocephalus may serve as a possible protective factor, in the way that patients without acute hydrocephalus at admission had a lower risk for later shunt placement. This suggestion is based on very few patients and in need of prospective validation. Subsequently, the most promising finding with SDASH application to our cohort was that none of the 38 patients with SDASH scores 0–1 was readmitted for shunting of chronic hydrocephalus during follow-up although 50% received shunts during initial hospitalization. Definite clinical findings which can safely exclude or identify patients at risk of delayed hydrocephalus would have clinical implications but must be confirmed in larger prospective studies with long term follow-up and strict criteria to define symptomatic hydrocephalus.

### Limitations and strengths

The study is limited by its retrospective design where uniform treatment is based on policy agreement among the treating physicians yet open to individual interpretation and variation. The 59% rate of shunt placement in our population was higher than the 31–40% [1, 7, 20] reported in comparable studies of EVD treated patients. This may reflect differences in clinical decision thresholds for EVD treatment and permanent shunt placement. Possibly the high shunting rate in patients with an EVD and acute hydrocephalus might diminish a statistical effect of the studied clinical factors.

Strengths include consecutive population-based inclusion with access to all relevant healthcare data. The main endpoint is shunting, which is based on identification of patients with hydrocephalus and physician’s assessment of need and benefit.

## Conclusion

We suggest that the decision to implant a permanent shunt in a population of patients with aSAH and an EVD is better based on individual assessment and clinical follow-up rather than any treatment algorithm or prediction score. Twenty (20) percent of patients who had acute hydrocephalus with initial successful discontinuation and discharge were readmitted for shunt insertion. With this high proportion, discharge must be made with an explicit follow-up strategy of post-discharge imaging to identify progressive hydrocephalus and allow shunt insertion before clinical deterioration.

## Data Availability

Data will be available upon reasonable request and only according to local regulations.

## Abbreviations

aSAH: Aneurysmal subarachnoid haemorrhage
BNI: Barrow Neurological Institute
CSF: Cerebrospinal fluid
CTA: Computed Tomography Angiography (CTA)
CT: Computer tomography
DSA: Digital Subtraction Angiography
EVD: External ventricular drain
GCS: Glasgow Coma Score
ICP: Intracranial pressure
MRI: Magnetic Resonance Imaging
ROC: Receiver Operating Characteristics
SDASH: Shunt Dependency in aSAH
VP: Ventriculoperitoneal
